# Phylogenetic Distribution of Secondary Metabolites in the Bacillus subtilis Species Complex

**DOI:** 10.1128/mSystems.00057-21

**Published:** 2021-03-09

**Authors:** Kat Steinke, Omkar S. Mohite, Tilmann Weber, Ákos T. Kovács

**Affiliations:** a Bacterial Interactions and Evolution Group, DTU Bioengineering, Technical University of Denmark, Kongens Lyngby, Denmark; b The Novo Nordisk Foundation Center for Biosustainability, Technical University of Denmark, Kongens Lyngby, Denmark; Wageningen University; University of California San Diego

**Keywords:** *Bacillus*, biosynthetic gene clusters, fengycin, iturin, phylogeny, plipastatin, secondary metabolite

## Abstract

Microbes produce a plethora of secondary (or specialized) metabolites that, although not essential for primary metabolism, benefit them to survive in the environment, communicate, and influence cell differentiation. Biosynthetic gene clusters (BGCs), responsible for the production of these secondary metabolites, are readily identifiable on bacterial genome sequences. Understanding the phylogeny and distribution of BGCs helps us to predict the natural product synthesis ability of new isolates. Here, we examined 310 genomes from the Bacillus subtilis group, determined the inter- and intraspecies patterns of absence/presence for all BGCs, and assigned them to defined gene cluster families (GCFs). This allowed us to establish patterns in the distribution of both known and unknown products. Further, we analyzed variations in the BGC structures of particular families encoding natural products, such as plipastatin, fengycin, iturin, mycosubtilin, and bacillomycin. Our detailed analysis revealed multiple GCFs that are species or clade specific and a few others that are scattered within or between species, which will guide exploration of the chemodiversity within the B. subtilis group. Surprisingly, we discovered that partial deletion of BGCs and frameshift mutations in selected biosynthetic genes are conserved within phylogenetically related isolates, although isolated from around the globe. Our results highlight the importance of detailed genomic analysis of BGCs and the remarkable phylogenetically conserved erosion of secondary metabolite biosynthetic potential in the B. subtilis group.

**IMPORTANCE** Members of the B. subtilis species complex are commonly recognized producers of secondary metabolites, among those, the production of antifungals, which makes them promising biocontrol strains. While there are studies examining the distribution of well-known secondary metabolites in *Bacilli*, intraspecies clade-specific distribution has not been systematically reported for the B. subtilis group. Here, we report the complete biosynthetic potential within the B. subtilis group to explore the distribution of the biosynthetic gene clusters and to reveal an exhaustive phylogenetic conservation of secondary metabolite production within *Bacillus* that supports the chemodiversity within this species complex. We identify that certain gene clusters acquired deletions of genes and particular frameshift mutations, rendering them inactive for secondary metabolite biosynthesis, a conserved genetic trait within phylogenetically conserved clades of certain species. The overview guides the assignment of the secondary metabolite production potential of newly isolated *Bacillus* strains based on genome sequence and phylogenetic relatedness.

## OBSERVATION

**FIG 1:**
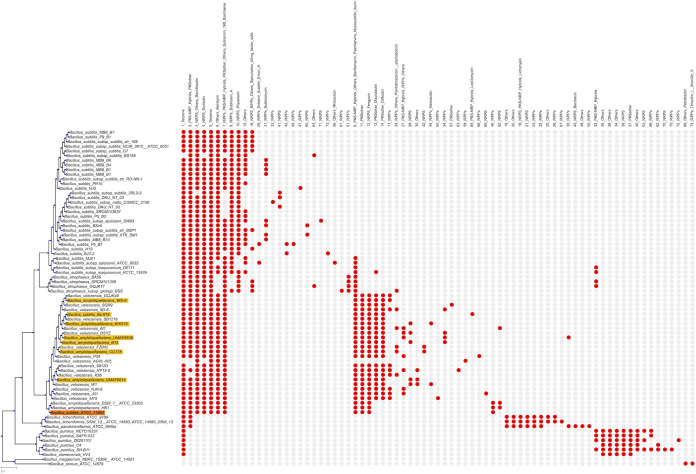
Phylogenetic tree reconstructed based on a multilocus sequence alignment of 30 genes with a modified version of autoMLST, using IQ-TREE and ultrafast bootstrapping with 1,000 replicates. B. cereus ATCC 14579 and B. megaterium NBRC 15308 were used as an outgroup. The presence-absence matrix of GCFs is visualized with a red dot indicating presence and a gray dot indicating absence. Figure S1 includes the complete tree. Strains with disagreements in NCBI and GTDB taxonomy are highlighted.

*Bacilli* can be isolated from various environments, such as the plant rhizosphere and the animal and human digestive systems, where secondary (or specialized) metabolites (SMs) play a pivotal role. The Bacillus subtilis group, which includes B. subtilis and its closely related species (Fig. 1), comprises common producers of bioactive SMs, such as antimicrobials and cytotoxic substances, empowering them for a range of industrial applications, including plant pathogen biocontrol (1, 2). Members of the B. subtilis group are producers of numerous well-known natural products, such as iturin, mycosubtilin, fengycin (FEN)/plipastatin (PPS), or bacillaene. Previous studies have globally reported the presence of known and novel biosynthetic gene clusters (BGCs) in *Bacillus* and related genera, highlighting the diverse potential of SM production in these bacteria (3–5). Additional reviews provide an overview of various SMs produced by these *Bacilli* (1, 6). Only recently, the species-level distribution of the corresponding BGCs in numerous coisolates from the B. subtilis group has been experimentally investigated (7).

10.1128/mSystems.00057-21.3 FIG S1 Phylogenetic tree and presence-absence matrix of different BGC families across the B. subtilis group. Download FIG S1, PDF file, 2.2 MB. Copyright © 2021 Steinke et al. 2021 Steinke et al. https://creativecommons.org/licenses/by/4.0/ This content is distributed under the terms of the Creative Commons Attribution 4.0 International license.

Here, we specifically expand previous studies by investigating patterns in all complete genomes of B. subtilis group as of July 2019 to dissect inter- and intraspecies diversity. Therefore, we examined the phylogenetic distribution of BGC families across 310 B. subtilis group genomes (see Data Set S1 in the supplemental material) by predicting BGCs with a modified version of antiSMASH v5.0 (8), clustering these into gene cluster families (GCFs) with BiG-SCAPE (9), and visualizing GCF distributions across a phylogenetic tree generated with autoMLST-derived scripts (10) (Fig. S1 and S2).

10.1128/mSystems.00057-21.1 DATA SET S1 Excel table with information on all the genomes, GTDB phylogeny, MLST genes, BGCs detected, and BiG-SCAPE-defined GCFs along with singleton BGCs. Download Data Set S1, XLSX file, 0.4 MB. Copyright © 2021 Steinke et al. 2021 Steinke et al. https://creativecommons.org/licenses/by/4.0/ This content is distributed under the terms of the Creative Commons Attribution 4.0 International license.

10.1128/mSystems.00057-21.4 FIG S2 Similarity network overview of all BGCs detected across B. subtilis group genomes visualized using Cytoscape (P. Shannon, A. Markiel, O. Ozier, N. S. Baliga, et al., Genome Res 13:2498–2504, 2003, https://doi.org/10.1101/gr.1239303). The nodes represent BGCs, and edges represent the similarity index calculated using BiG-SCAPE. The nodes are colored based on the species of the genome (see the color key). The labeled nodes are the known BGCs from the MIBiG database. Note that MIBiG has two different versions for the BGCs bacillibactin (BGC0000309.1 [v1] and BGC0001185.1 [v2]) and bacilysin (BGC0001184.1 [v1] and BGC0000888.1 [v2]). Download FIG S2, PDF file, 1.7 MB. Copyright © 2021 Steinke et al. 2021 Steinke et al. https://creativecommons.org/licenses/by/4.0/ This content is distributed under the terms of the Creative Commons Attribution 4.0 International license.

The phylogenetic tree was reconstructed based on a multilocus sequence alignment of 30 conserved single-copy genes (Fig. 1; Fig. S1), generally reflecting NCBI taxonomy, but with certain disagreements in the *B. velezensis* and *B. amyloliquefaciens* clades (highlighted in Fig. 1 and below).

The 3,643 BGCs identified using antiSMASH v5.0 (8) were assigned into 75 GCFs and 62 singletons with BiG-SCAPE (9) (Fig. S2); GCFs were subsequently mapped to the tree (Fig. 1; Fig. S1). Only one predicted GCF, coding a terpene (sesquarterpene), was found in nearly all strains, while another, a predicted nonribosomal peptide synthetase (NRPS)/polyketide synthase (PKS) hybrid, was found in most species except *B. pumilus* and *B. xiamenensis*. Other widespread GCFs are bacillibactin, surfactin, and bacilysin. Bacillaene and sublancin 168 families were found in most species, except B. licheniformis, B. paralicheniformis, B. pumilus, and B. xiamenensis; however, there are two gaps seemingly following clade boundaries in B. subtilis. A similar gap in distribution occurs in bacilysin, which is absent in B. spizizenii and B. atrophaeus. No correlation was identified between the determined BGC number of each strain and the source of isolation (e.g., rhizosphere, soil, food, or environment) (Data Set S1).

Such apparently clade-linked patterns in the absence or presence of GCFs were common, and many GCFs were distributed according to phylogeny. This occurred in both individual species and clades spanning multiple species. For example, a clade-specific GCF, lichenysin, was identified only in B. licheniformis and *B. paralicheniformis*. The distribution of the highly similar lipopeptides fengycin and plipastatin also followed clade boundaries, with fengycin in B. velezensis and B. amyloliquefaciens, whereas plipastatin was found in B. subtilis and B. atrophaeus. As previously reported (11), GCFs for rhizocticin but not plipastatin were found in *B. spizizenii*, supporting the biosynthetic distinctness of this clade.

Other examples of clusters almost or entirely limited to one species in the tree included bacitracin, which was present in all examined *B. paralicheniformis* genomes, as well as difficidin and macrolactin, both found in most *B. velezensis* strains (though macrolactin was also present in single isolates of other species). Certain species-specific GCFs were found dispersedly; for instance, the B. subtilis-specific subtilomycin was apparently linked to particular clades within the species or the ribosomally synthesized and posttranslationally modified peptide-coding 33_RiPP and 49_RiPP families, which were also species specific. Additionally, some families appeared in multiple clades but in a clade-linked pattern (17 ribosomally synthesized and posttranslationally modified peptides [RiPPs] in *B. velezensis*), while others were missing in one or more clades (15_Others in B. subtilis).

Finally, other GCFs appeared more scattered within a species, with no evident link to an individual clade, such as the 42_NRPS GCF in *B. velezensis* (Fig. S1). Only a few GCFs (e.g., 39_RiPPs), appeared scattered across the entire tree without a noticeable link to particular clades. Horizontal gene transfer (HGT), in accordance with the natural competence of B. subtilis (6), might drive the scattered patterns and random occurrences of GCFs outside key species. For instance, the 42_NRPS family contains the *nrs* cluster of *B. velezensis* FZB42, previously suggested to be acquired via HGT (12).

Next, we compared the genetic variations within particular GCFs and investigated the phylogenetic relationship among these variants, selecting families that code for important *Bacillus* SMs, namely fengycin, plipastatin, iturin, bacillomycin, and mycosubtilin.

**FIG 2:**
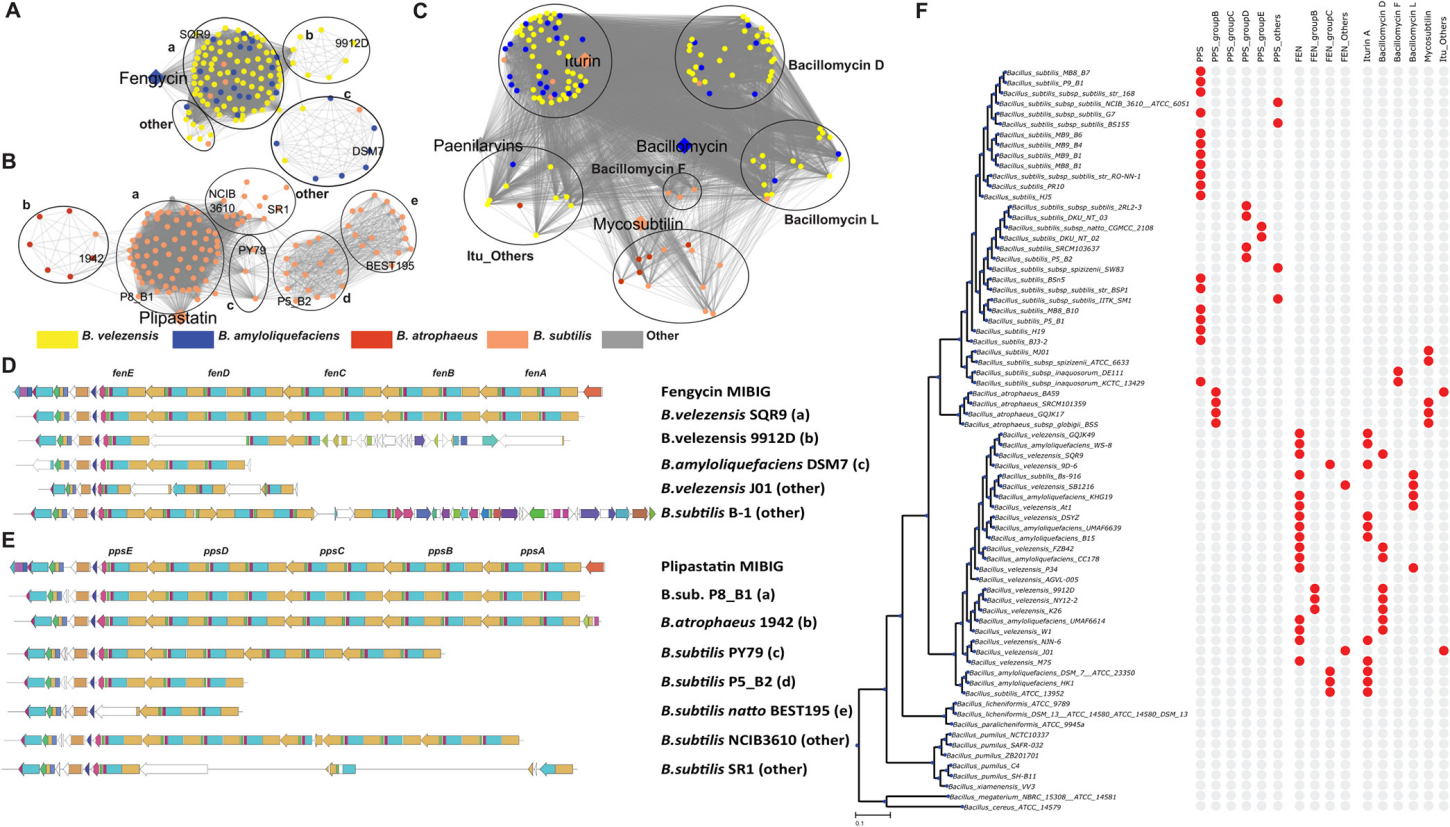
Comparison of plipastatin/fengycin/iturin families of BGCs. (A to C) Similarity networks representing the plipastatins, fengycins, and iturinic lipopeptide GCFs, respectively. The different colors represent different species of *Bacillus.* Iturinic lipopeptides are grouped based on amino acid specificity predictions instead of BiG-SCAPE-generated similarity index (Table S1). (D to E) Selected clusters are shown from different groups of plipastatins and fengycins, respectively. The detailed genetic structures of all incomplete BGC families can be found in Fig. S3. (F) The phylogenetic distribution of different groups of BGCs is presented across selected genomes. For a complete list of all genomes and different groups of BGCs, see Fig. S4.

A total of 127 BGCs were part of the similarity network with BGCs for plipastatin, a biodegradable fungicide (1). Based on the similarity network, these BGCs were placed into 6 groups: PPS, PPS groups B to E, and PPS_others (Fig. 2; Data Set S2). Plipastatins are mostly observed in the B. subtilis strains, with the exception of group B BGCs found in B. atrophaeus. We found that 71 BGCs from group PPS and 7 BGCs from group B had the complete BGC for plipastatin (*ppsA* to *ppsE*). In contrast, groups C, D, E, and “others” had BGCs missing up to three biosynthetic genes (BGs) (Fig. S3), consistent with experimental data demonstrating a lack of plipastatin production in B. subtilis natto BEST195 (13) and B. subtilis P5_B2 (7) (Fig. 2). A similar deletion of BGs was found in several other strains. Interestingly, these strains are phylogenetically close to each other, suggesting such deletions being conserved within a single clade (Fig. 2F; Fig. S4). Additionally, in plipastatin BGCs of group E, gene *ppsE* appeared to have missing domains (Fig. S5). Investigation of the nucleotide sequences of *ppsE* gene homologs revealed a deletion at position 232 of the reference *ppsE* gene across all 16 members of group E, leading to a frameshift mutation causing alternative protein sequence translation that lacks the respective functional domains (Fig. S5). This frameshift was present in multiple strains isolated from distinct geographic locations (Data Set S2) but belonging to the same phylogenetic clade, suggesting an evolutionarily conserved frameshift in the *ppsE* gene that may lead to loss of function.

10.1128/mSystems.00057-21.2 DATA SET S2 Excel table with information on BGCs from families encoding fengycins, plipastatins, and iturinic lipopeptides. Download Data Set S2, XLSX file, 0.1 MB. Copyright © 2021 Steinke et al. 2021 Steinke et al. https://creativecommons.org/licenses/by/4.0/ This content is distributed under the terms of the Creative Commons Attribution 4.0 International license.

10.1128/mSystems.00057-21.5 FIG S3 Genetic structure variations in partial BGCs of the plipastatin and fengycin families. Download FIG S3, PDF file, 2.4 MB. Copyright © 2021 Steinke et al. 2021 Steinke et al. https://creativecommons.org/licenses/by/4.0/ This content is distributed under the terms of the Creative Commons Attribution 4.0 International license.

10.1128/mSystems.00057-21.6 FIG S4 Phylogenetic tree distribution of the presence of different plipastatins, fengycins, and iturinic lipopeptides and the presence of the frameshift mutation *sfp* gene across 310 genomes of *Bacillus* sp. Download FIG S4, PDF file, 1.1 MB. Copyright © 2021 Steinke et al. 2021 Steinke et al. https://creativecommons.org/licenses/by/4.0/ This content is distributed under the terms of the Creative Commons Attribution 4.0 International license.

10.1128/mSystems.00057-21.7 FIG S5 Conserved frameshift in the *ppsE* gene across strains harboring group E plipastatin BGCs. (A) BGCs from group E of plipastatins with missing domains in the *ppsE* gene. (B) Part of the phylogenetic tree, including plipastatin group E strains (see the complete tree in Fig. S4). (C) Nucleotide sequence alignment with a conserved deletion at position 232 that led to a frameshift. The nucleotide fasta sequences of these selected homologs were aligned using MUSCLE (R. C. Edgar, Nucleic Acids Research 32:1792–1797, 2004, https://doi.org/10.1093/nar/gkh340), and the alignments were visualized using Jalview (M. Clamp, J. Cuff, S. M. Searle, G. J. Barton, Bioinformatics 20:426–427, 2004, https://doi.org/10.1093/bioinformatics/btg430). Download FIG S5, PDF file, 1.8 MB. Copyright © 2021 Steinke et al. 2021 Steinke et al. https://creativecommons.org/licenses/by/4.0/ This content is distributed under the terms of the Creative Commons Attribution 4.0 International license.

10.1128/mSystems.00057-21.10 TABLE S1 Amino acid specificity prediction-based groups of iturin-like BGCs. Download Table S1, PDF file, 0.02 MB. Copyright © 2021 Steinke et al. 2021 Steinke et al. https://creativecommons.org/licenses/by/4.0/ This content is distributed under the terms of the Creative Commons Attribution 4.0 International license.

The fengycin family network contained 123 BGCs from *B. velezensis* and *B. amyloliquefaciens* species, in addition to 5 isolates likely misclassified as “B. subtilis.” Based on our multilocus sequence assay (MLSA) data, these should be assigned as *B. velezensis* (Data Set S2; Fig. S4). The fengycin BGCs could be divided into four groups, with 96 BGCs containing all BGs (*fenA* to *fenE*). BGCs from groups B, C, and others contained incomplete BGCs, with up to three of the BGs missing (Fig. S3). The strains harboring these incomplete fengycin BGCs were also phylogenetically close, similar to plipastatins, suggesting that these deletions were conserved within a single clade (Fig. 2F; Fig. S4). As noted above for the *ppsE* gene, many phylogenetically close strains harboring group B of fengycin contained a frameshift at positions 3126 to 3127 of the *fenD* gene, suggesting a possible evolutionary trait of the clade (Fig. S6). Again, these frameshift mutations result in the translation of an alternative protein sequence lacking the functional domains of FenD.

10.1128/mSystems.00057-21.8 FIG S6 Conserved frameshift in the *fenD* gene across strains harboring group B fengycin BGCs. (A) BGCs from group B of fengycins with missing domains in the *fenD* gene. (B) Part of the phylogenetic tree, including fengycin group B strains (see the complete tree in Fig. S4). (C) Nucleotide sequence alignment with a conserved deletion at positions 3126 to 3127 that led to a frameshift. Download FIG S6, PDF file, 1.1 MB. Copyright © 2021 Steinke et al. 2021 Steinke et al. https://creativecommons.org/licenses/by/4.0/ This content is distributed under the terms of the Creative Commons Attribution 4.0 International license.

Unlike with the above, sequence similarity alone could not divide the 141 iturin-like BGCs into distinct groups due to conserved BG sequences. These BGs differ only in the individual amino acid substrate specificities leading to the production of diverse lipopeptides, like iturin A, bacillomycin D-F-L, and mycosubtilin (14), with different levels of bioactivity (6). Therefore, the antiSMASH-predicted amino acid substrate specificities for all NRPS adenylation domains were used to group the BGCs into iturin A, bacillomycin D-F-L, mycosubtilin, and “others” that have less than the 7 amino acid substrates that are typical for iturins (Table S1; Data Set S2). Mapping these data onto the phylogenetic tree revealed that each group is conserved in closely related strains. The mycosubtilin group was detected in B. atrophaeus and in some B. subtilis and *B. spizizenii* strains, and bacillomycin was detected in three strains of *B. inaquosorum*, whereas iturin A, bacillomycin D, and bacillomycin F were spread across different *B. velezensis* and *B. amyloliquefaciens* isolates, confirming the previously proposed species- and strain-level presence of iturinic lipopeptides (14).

The lack of SM production, specifically surfactin, in domesticated strains of B. subtilis has previously been connected to a frameshift mutation in the *sfp* gene, coding for a 4-phosphopantetheinyl transferase, which transfers the essential phosphopantetheine prosthetic group to the surfactin NRPS (15). This frameshift mutation in the *sfp* gene (all identical to previously reported positions) was detected in only 13 of 260 genes, all belonging to closely related laboratory strains and an additional B. subtilis isolate (Data Set S2; Fig. S7), suggesting that the inactivation of lipopeptide production in natural *Bacillus* isolates is not as common as might have been expected based on the laboratory observations.

10.1128/mSystems.00057-21.9 FIG S7 Conserved frameshift mutation of the *sfp* gene in 14 genomes of B. subtilis. Download FIG S7, PDF file, 2.3 MB. Copyright © 2021 Steinke et al. 2021 Steinke et al. https://creativecommons.org/licenses/by/4.0/ This content is distributed under the terms of the Creative Commons Attribution 4.0 International license.

Our detailed BGC comparison identified variations in particular GCFs to be phylogenetically conserved but also revealed that particular GCFs were clade rather than species specific. Therefore, our study improves upon the previous systematic descriptions of BGCs within the *Bacillus* genus (3–5) by providing a more detailed, species-level examination of the distributions and features of BGCs within strains belonging to the environmentally important B. subtilis group. Such phylogenetic correlation of different BGC groups and particular frameshifts suggest evolutionary relationships among production capabilities of *Bacillus* strains. Therefore, our workflow, combining comparative analysis of BGCs and phylogenetic relationships, revealed how a particular BGC evolves within a species. This knowledge, and closer examination of the exceptions, may guide the selection of specific strains as antimicrobial producers within underexplored groups of SM producers.

### Genome selection.

Initially, all genomes of *B. amyloliquefaciens*, B. atrophaeus, B. licheniformis, *B. paralicheniformis*, *B. pumilus*, B. subtilis, *B. velezensis*, *B. xiamenensis*, and a few related *Bacillus* sp. strains with assembly status “complete” or “chromosome” publicly available from the NCBI in July 2019 were selected. Additionally, the type strains of B. cereus and B. megaterium were included as outgroups. The strain list was then curated to remove duplicates. Further, the genomes of engineered B. subtilis and strains were removed (B. subtilis BEST7613, B. subtilis delta6, B. subtilis IIG-Bs27-47-24, B. subtilis PS38, and B. subtilis PG10, as described in reference 16, as well as B. subtilis BEST7003, B. subtilis QB5413, B. subtilis QB5412, B. subtilis QB928, and B. subtilis WB800N). After preliminary tree reconstruction, B. subtilis HDZK-BYSB7 was found to group with B. cereus rather than the other B. subtilis strains and was therefore removed; it has since been reclassified as B. anthracis. Initial examination of results also found BGCs to be split by the origin in *B. velezensis* Hx05; for ease of analysis, this strain was therefore dropped. Subsequently, *B. velezensis* AGVL-005 was found to contain many frameshifted proteins; however, it was retained. A further 13 in-house genomes of B. subtilis and one of B. licheniformis (17) were included. This led to a final count of 310 genomes.

### Genome acquisition and strain name annotation.

Genomes were downloaded in the NCBI GenBank format with the ncbi-acc-download tool (https://github.com/kblin/ncbi-acc-download). As many of the GenBank entries did not contain strain information in the “Source” or “Organism” features, which are required by the autoMLST and BiG-SCAPE tools to distinguish the individual strains, the Python script rename_strainless_organisms.py (found in the tree and matrix construction pipeline [see below]) was employed to transfer strain information from the “strain” field to these fields.

### Genome mining.

In a first step, all downloaded genomes were initially mined for SMs with antiSMASH 5.0 (8). antiSMASH collapses gene clusters that are in close proximity, such as the iturin and fengycin clusters in *Bacilli*, into a single biosynthetic “region.” A modified version of antiSMASH (https://github.com/KatSteinke/dmz-antismash) that contains the additional functionality to split known clusters at a user-defined gene, resulting in two independent “regions,” was developed. In all other respects, this version of antiSMASH is identical to antiSMASH 5.0.0. The modified version of antiSMASH was run as an antiSMASH fast run with the default parameters. The genes selected to split between adjacent clusters were *dacC* and *yngH* for the plipastatin/fengycin clusters and *yxjF* and *xynD* for the iturin clusters. For assigning the plipastatin/fengycin boundary genes, homologs from several species were selected to reflect species variations: *dacC* homologs from *B. velezensis*, B. subtilis, *B. amyloliquefaciens*, and B. atrophaeus and *yngH* homologs from B. subtilis and B. atrophaeus. These were selected so that the cut would yield the intersection of both clusters as found on MIBiG, from *dacC* to *yngH*, as other boundaries led to incorrect splits, either failing to cut the cluster or cutting it twice. The genes are identified by a BLAST search in the examined genome, with coverage and identity of at least 90% each needed for identification. During this step, errors in the GenBank file of B. licheniformis PB3 (accession no. NZ_CP025226.1) were detected, as they caused subsequent errors in antiSMASH; the erroneous portions, CXG95_RS00005 and CXG95_RS00010, were consequently deleted.

### GCF identification and clustering.

We used BiG-SCAPE (9) at default settings to identify families of homologous gene clusters present in multiple species (gene cluster families [GCFs]). In order to automatically identify any known compounds, reference clusters from the MIBiG database (18) were included in the networking analysis. Singleton clusters were not returned. As this produced almost exclusively GCFs split along species lines, even for compounds known to be found in all species, connected components were identified with the NetworkX library (19) using an approach similar to that in reference 20. However, as BiG-SCAPE was left at default options, duplicated entries were later merged.

### Tree building.

For getting a highly resolved phylogeny of the closely related *Bacillus* strains, maximum-likelihood trees were constructed with a pipeline based on autoMLST (10) and by using autoMLST defaults to the greatest extent. We introduced a modification to autoMLST that skipped the automated search and inclusion of similar genomes and thus processed only the supplied genomes. Subsequently, the pipeline identified all conserved single-copy genes from these genomes. Additionally, the gbk2sqldb.py script in autoMLST, which was employed in the pipeline, was patched to use the same hmm database (reducedcore.hmm) as the main automlst.py script. The modified version, including reducedcore.hmm, is available at https://github.com/KatSteinke/automlst-simplified-wrapper.

Both for the short tree shown in Fig. 1 and the full tree (Fig. S1), analysis with this pipeline yielded 30 single-copy/housekeeping genes for each tree; however, not all of these were identical between the trees. For generating the multilocus alignment, each individual gene was aligned with MAFFT (21) and the alignment trimmed using trimAl (22); then, all alignments were concatenated. As in autoMLST, the tree was generated with IQ-TREE (23), using Ultrafast Bootstrap (24) with 1,000 replicates.

The resulting tree was rerooted in ETE3 (25) during the visualization step, using B. megaterium NBRC 15308 and B. cereus ATCC 14579 as an outgroup. During this process, it was found that the GenBank file of B. subtilis subsp. *subtilis* NCD-2 had been excluded from the tree because it lacked gene annotations; thus, it was annotated with Prokka.

In our global analysis of all 310 genomes, we identified a total of 28 strains whose genome-based taxonomy conflicts with their assigned species names (Fig. S1; Data Set S1). Based on our analysis, in line with the recently released genome-based taxonomy in GTDB (26, 27), these strains should be designated *B. velezensis* or *B. amyloliquefaciens*, respectively. Additionally, strains designated B. subtilis subsp. *inaquosorum* and B. subtilis subsp. *spizizenii* by NCBI form their own clades, consistent with their recent promotion to species status (11). The tree thus appears to reflect genome-based taxonomy well.

### Absence/presence matrix.

We established an automated pipeline for the tree and matrix construction pipeline that combined the individual steps of the analysis. The script for this pipeline takes as arguments the location of a base directory in which analysis results are to be placed, the location of a file listing accession numbers to be downloaded, the name of the final tree to be output, and optional outgroups to be used. It creates all the files and directories necessary for the subsequent analysis (see below). The script can be downloaded at https://github.com/KatSteinke/AbsPresTree.

From the connected component GCFs, a matrix-counting occurrence of each GCF in each strain was computed. GCFs were subsequently clustered according to their occurrence in each strain using SciPy’s clustering package (28); hierarchical clustering was performed. Subsequently, the absence/presence matrix was reordered to reflect the clustering of GCFs.

It must be noted, however, that the connected-component GCFs are based on the placement of gene clusters in a network, and even incomplete or inactive clusters may be included if they pass the threshold for clustering. The tree and matrix were visualized in ETE3 using ETE3’s clustering module. Subsequently, matrix columns were manually arranged to follow the phylogeny of the strains primarily represented per column.

### Variations within particular GCFs.

Based on the similarity networks of the plipastatin and fengycin GCFs, we created groups within a GCF. The fengycin GCF was split into four groups, and the plipastatin GCF was split into six groups. The genetic structure variations among groups with few missing BGs are shown in Fig. S3. The genes *ppsE* and *fenD* from plipastatin group E and fengycin group B, respectively, are further selected for multiple-sequence alignment (Fig. S5 and S6). For the iturinic lipopeptide GCF, substrate specificities of the A domain were collected from antiSMASH annotations. Based on the individual amino acid specificities, the BGCs from this GCF are further classified into iturin A, bacillomycin D, F, and L, and mycosubtilin (Table S1). In addition to analyzing GCF variation, we aligned nucleotide sequences of the *sfp* gene, coding for 4-phosphopantetheinyl transferase, from 260 BGCs of the surfactin family (Data Set S2). A frameshift mutation previously known to disrupt *sfp* function was detected across 14 of the 260 genes (Fig. S7). We generated a presence-absence matrix where the rows represent 310 genomes and the columns represent groups of plipastatins (PPS, PPS groups B to E, PPS others), fengycins (FEN, FEN groups B and C, and FEN others), iturin A, bacillomycin D, F, and L, mycosubtilin, and *sfp* gene frameshift mutation (Fig. S4). The presence-absence matrix is visualized against the tree to understand the evolutionary aspects of GCF variation. The scripts used to analyze the variations in GCF can be downloaded at https://github.com/OmkarSaMo/GCF_variation_Bacillus.

### Data availability.

The data with NCBI accession IDs and information on all detected gene clusters are available in Data Sets S1 and S2. Code used to generate the data is available at https://github.com/KatSteinke/AbsPresTree. The script used to analyze the variations in GCF is available at https://github.com/OmkarSaMo/GCF_variation_Bacillus.

## Supplementary Material

Reviewer comments
