# Supplementary figures and images for: Phylogenetic Distribution of Secondary Metabolites in the Bacillus subtilis Species Complex
Source: mSystems. 2021 Mar 9;6(2):e00057-21. doi: 10.1128/mSystems.00057-21 (PMC8546965; doi:10.1128/mSystems.00057-21)

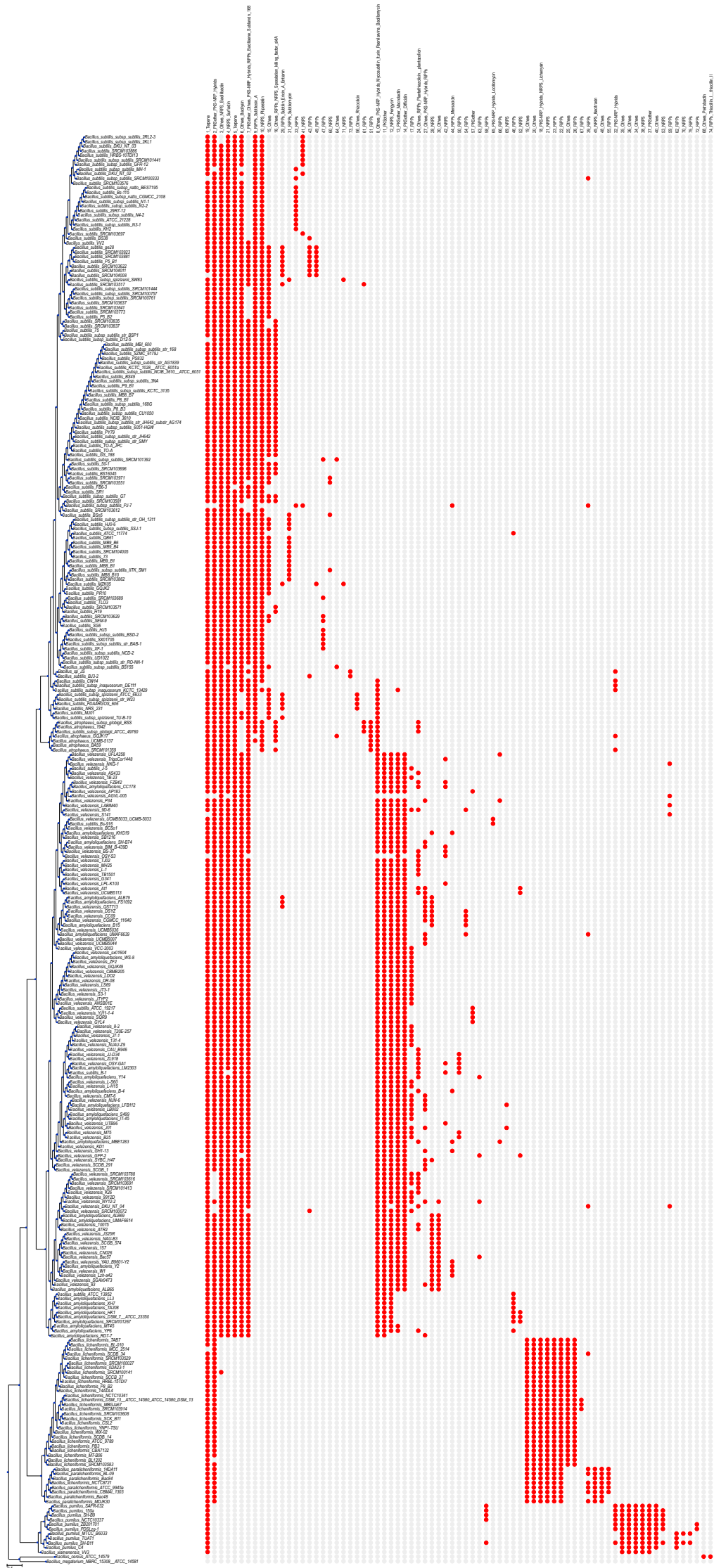

Supplement: FIG S1 [file msystems.00057-21-sf001.pdf]

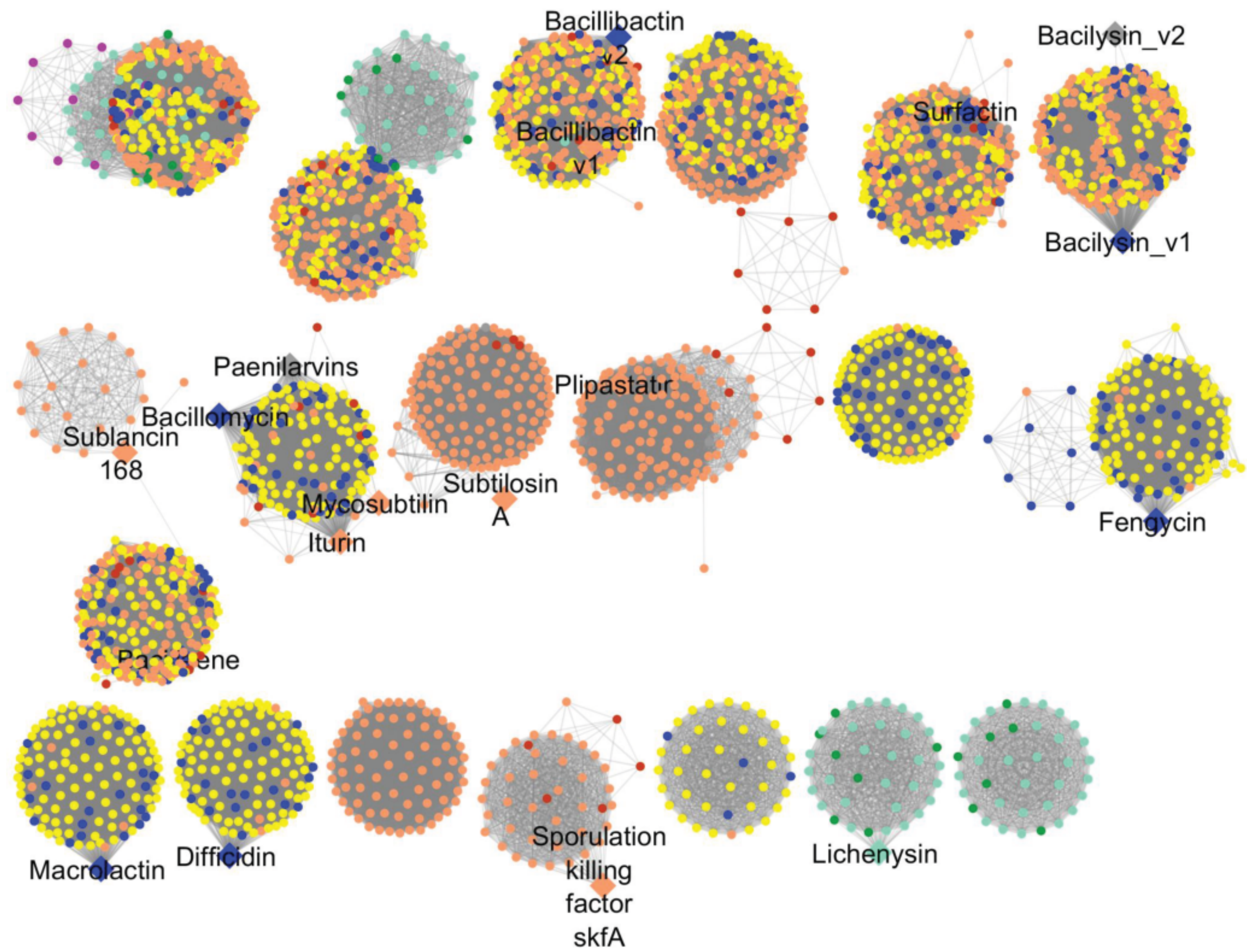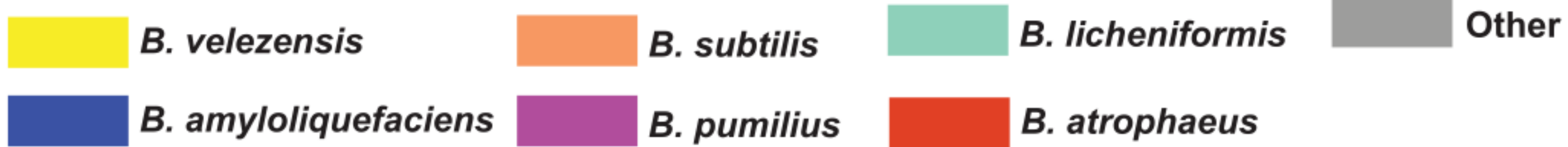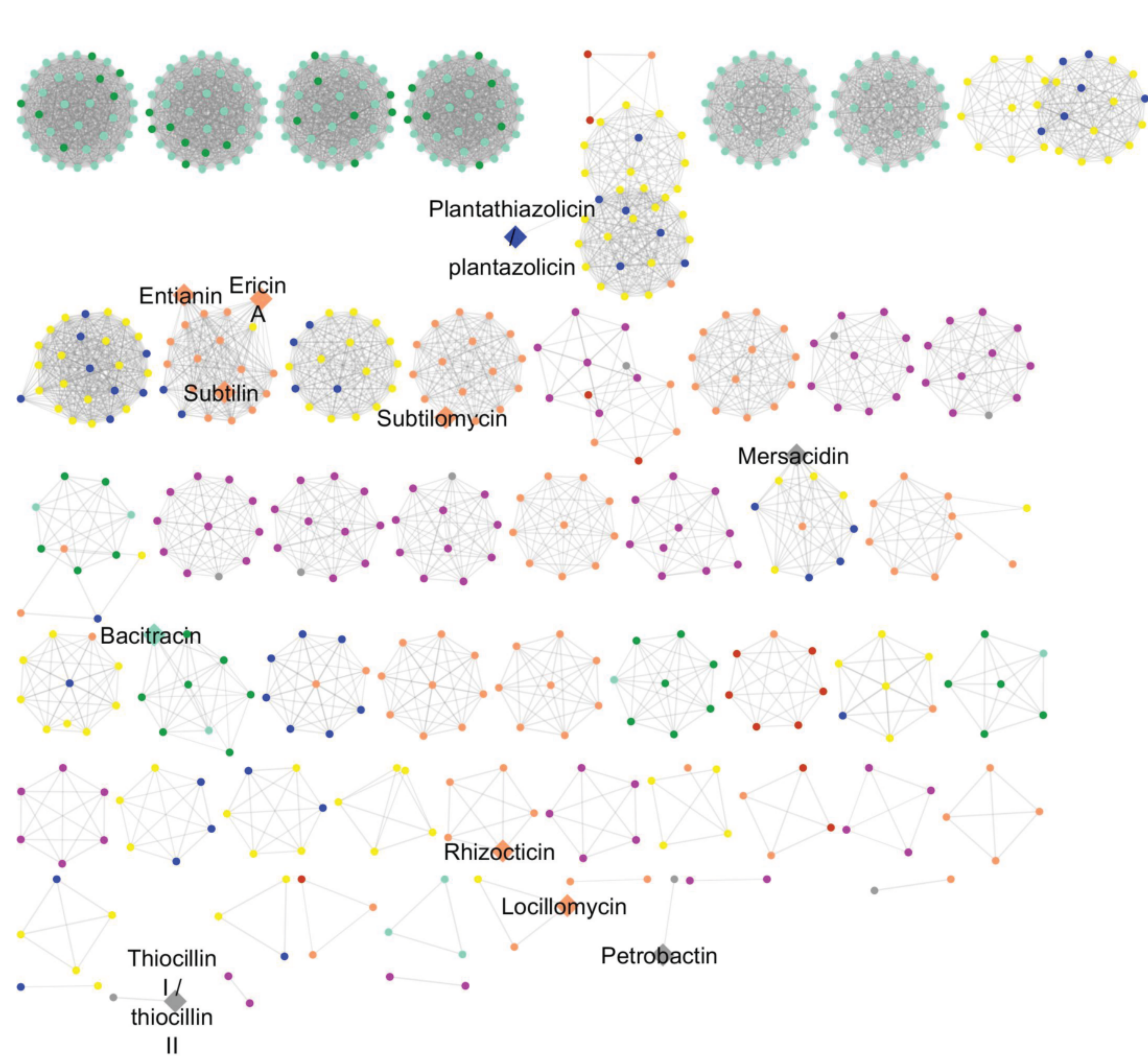

Supplement: FIG S2 [file msystems.00057-21-sf002.pdf]

A

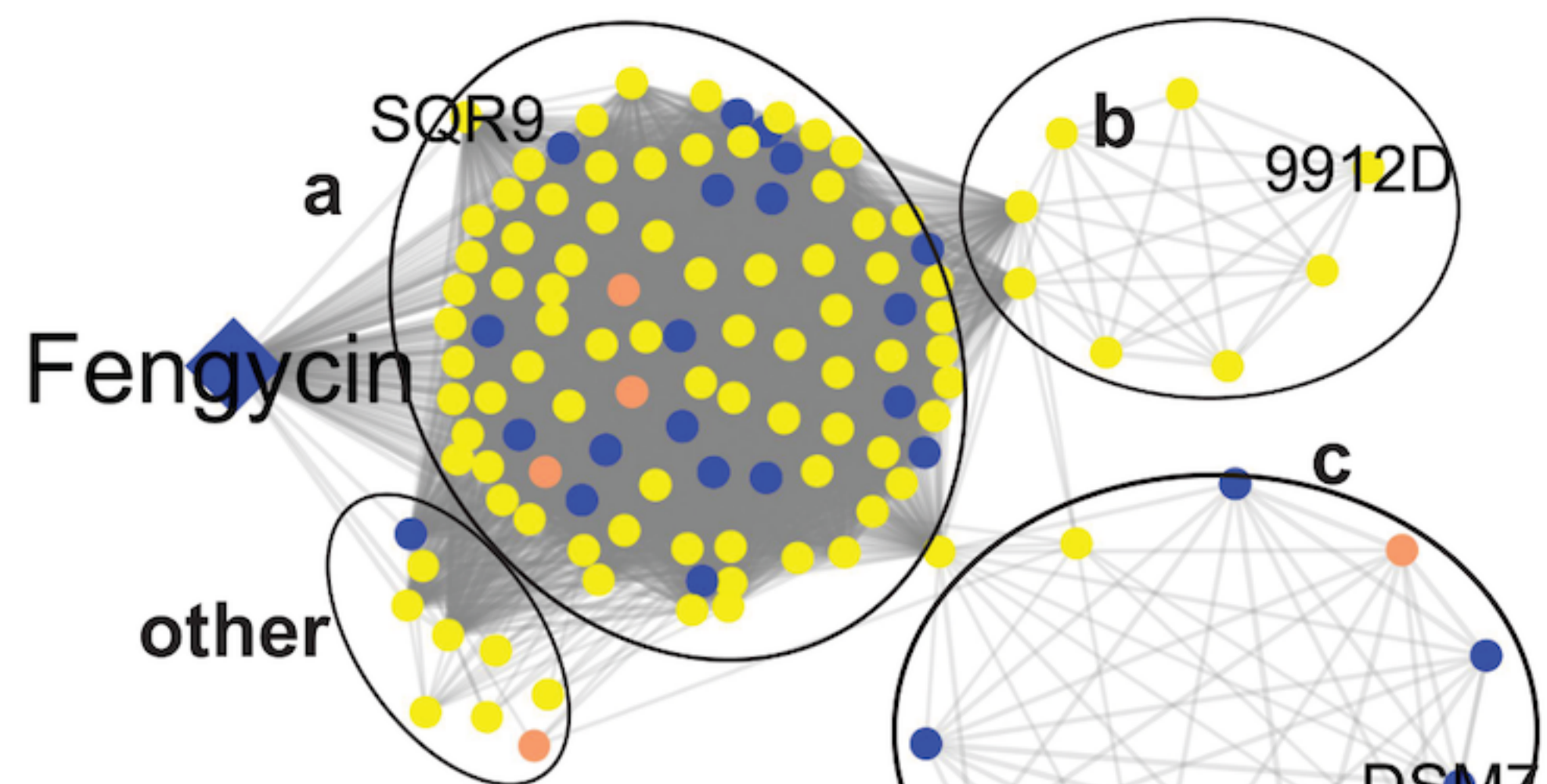

B

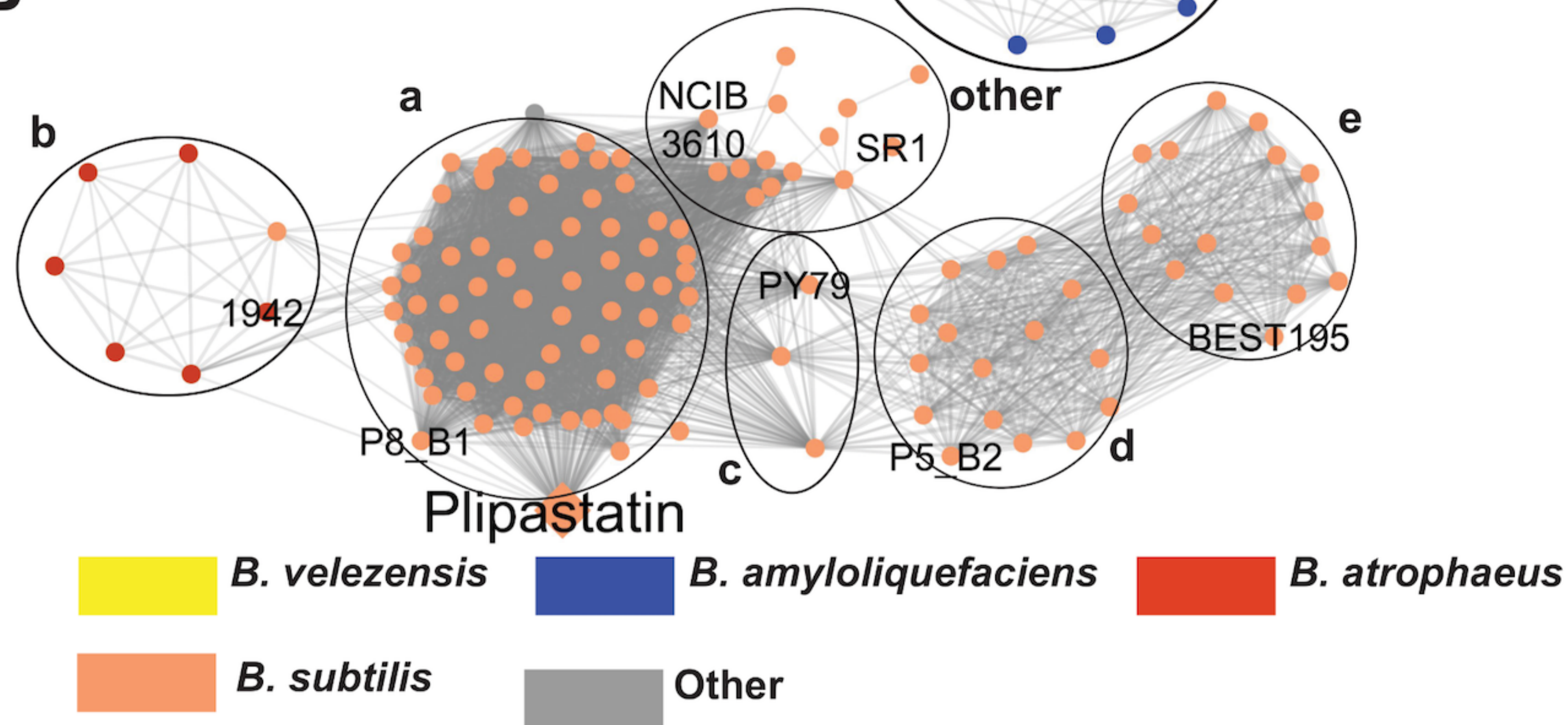

C

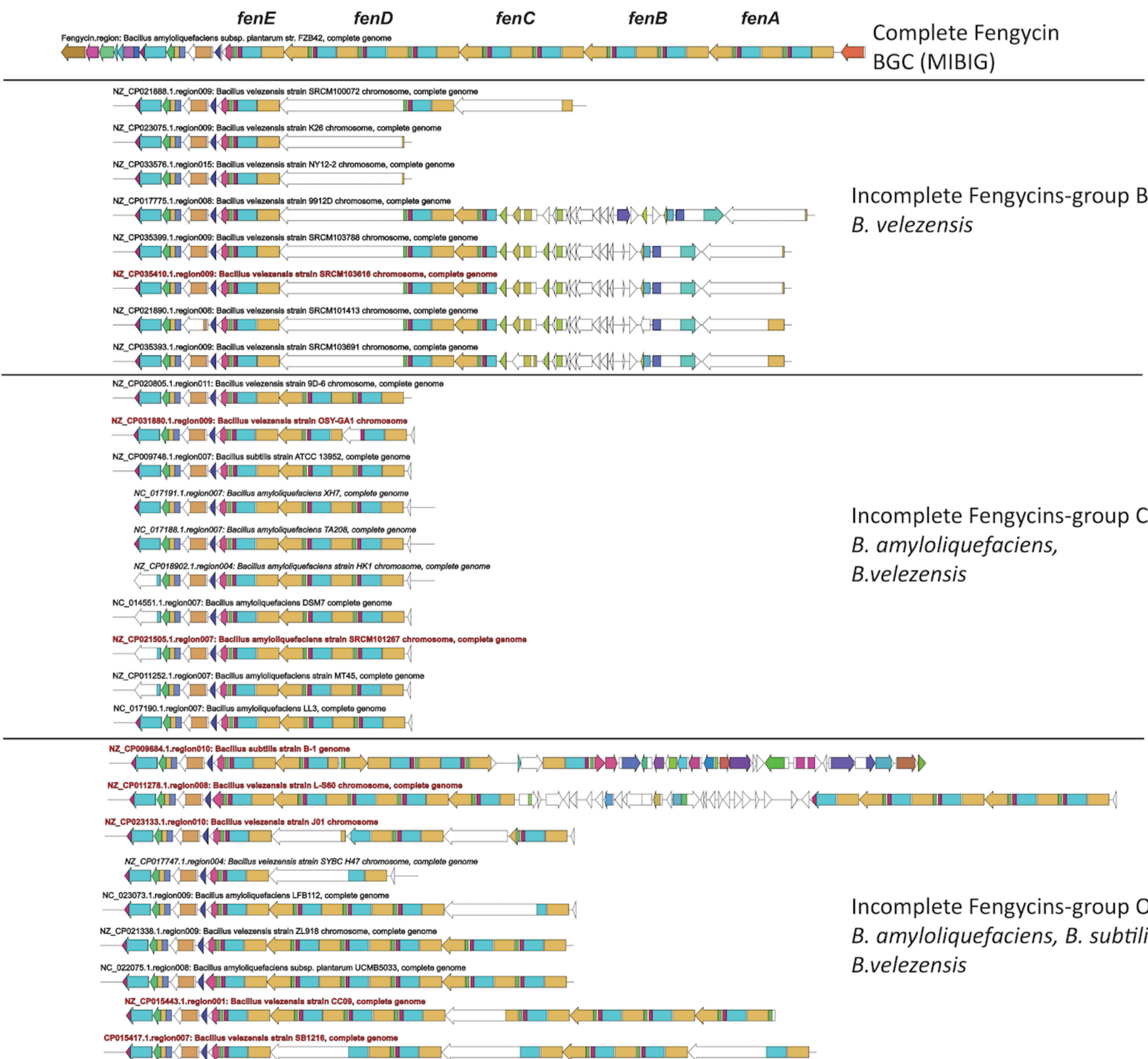

D

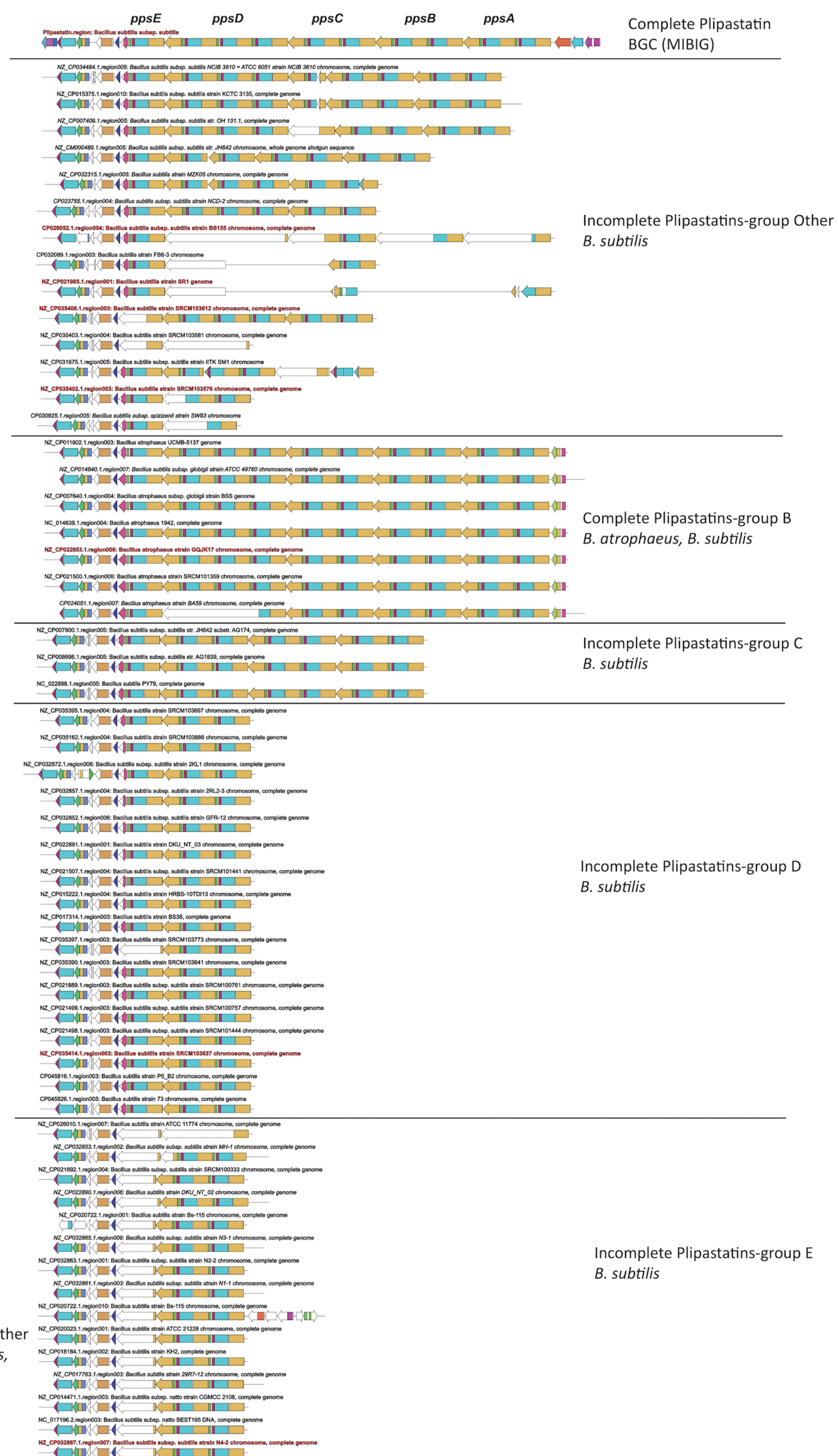

Supplement: FIG S3 [file msystems.00057-21-sf003.pdf]

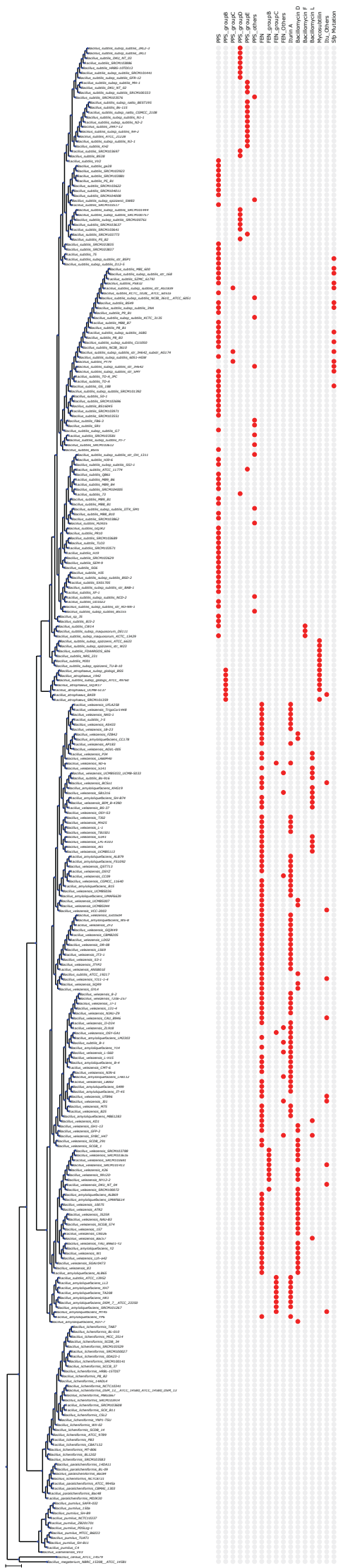

Supplement: FIG S4 [file msystems.00057-21-sf004.pdf]

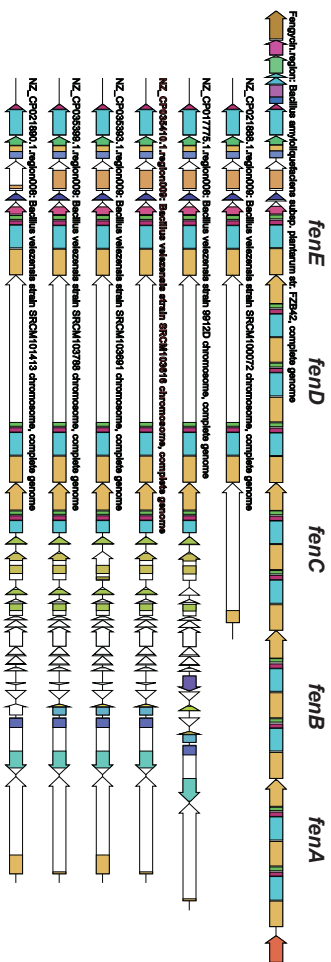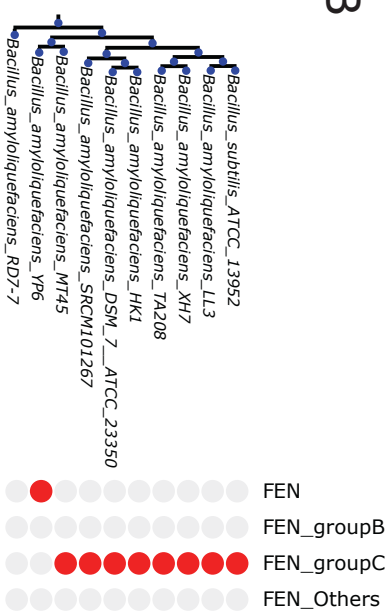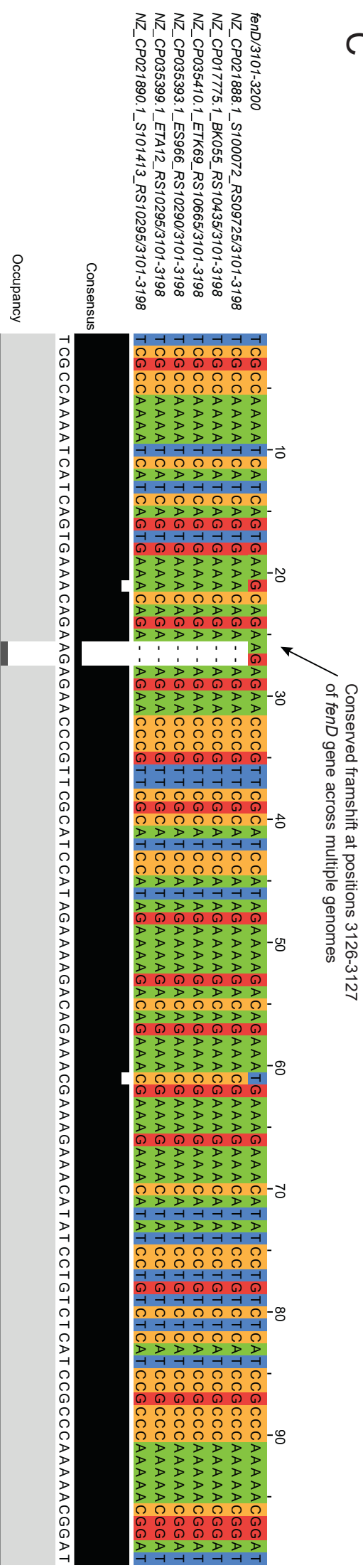

Supplement: FIG S6 [file msystems.00057-21-sf006.pdf]

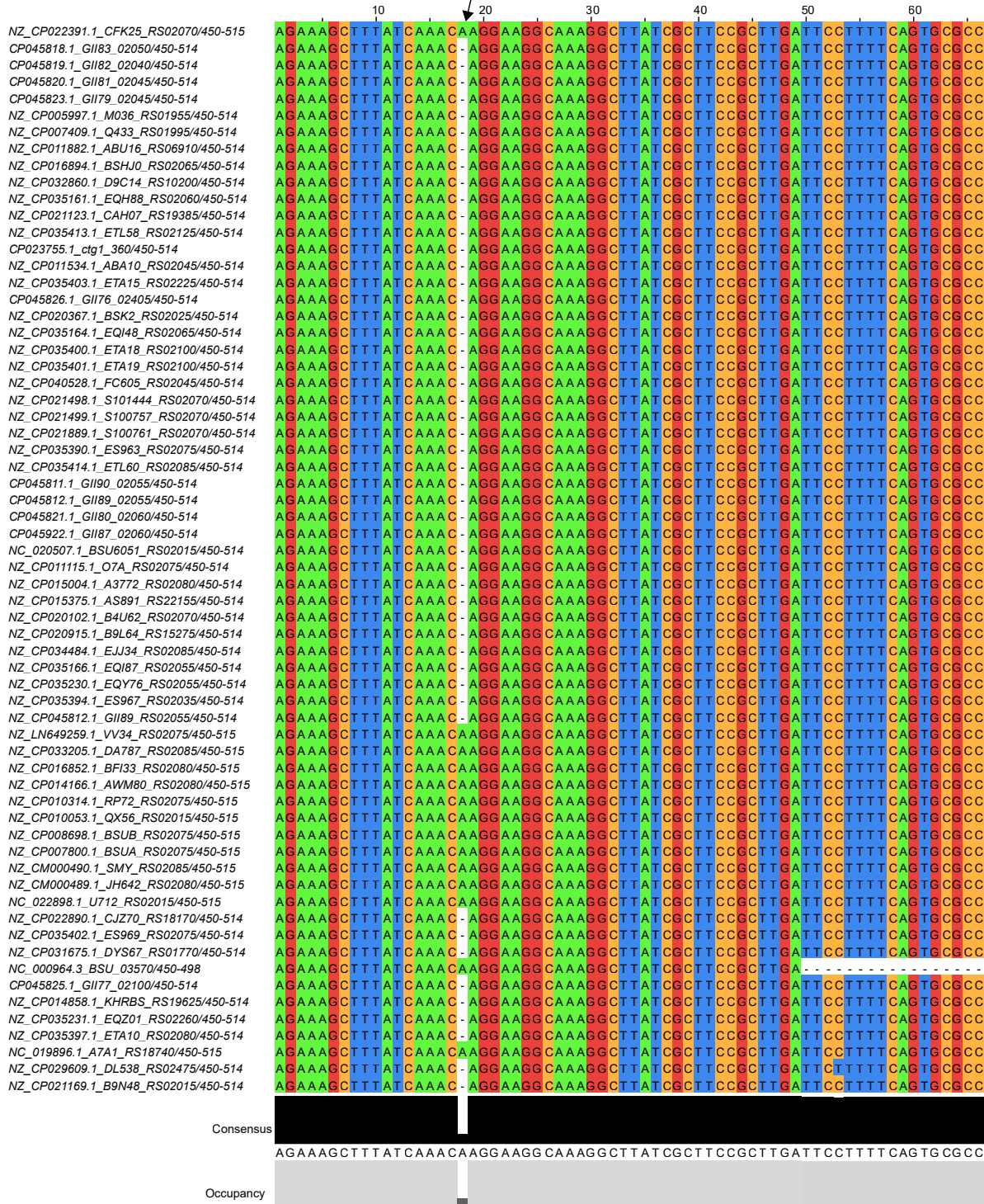

Supplement: FIG S7 [file msystems.00057-21-sf007.pdf]
