# Supplementary material for: Phylogenetic Distribution of Secondary Metabolites in the Bacillus subtilis Species Complex
Source: mSystems. 2021 Mar 9;6(2):e00057-21. doi: 10.1128/mSystems.00057-21 (PMC8546965; doi:10.1128/mSystems.00057-21)
Supplement: TABLE S1 [file msystems.00057-21-st001.pdf]

Table S1: Amino acid specificity prediction-based groups of iturinic lipopeptide BGCs

Group of BGC	AA prediction sequence	*B. subtilis*	*B. velezensis*	*B. amyloliquefaciens*	*B. atrophaeus*
Iturin A	ser,asn,pro,gln,asn,tyr,asn,fatty_acid,mal	3	42	19	0
Bacillomycin D	thr,ser,glu,pro,asn,tyr,asn,fatty_acid,mal	1	28	6	0
Bacillomycin F	thr,asn,pro,gln,asn,tyr,asn,fatty_acid,mal	3	0	0	0
Bacillomycin L	thr,ser,glu,ser,asn,tyr,asn,fatty_acid,mal	1	14	2	0
Mycosubtilin	asn,ser,pro,gln,asn,tyr,asn,fatty_acid,mal	7	0	0	5
Incomplete 1	asn,tyr,asn,fatty_acid,mal	0	2	0	0
Incomplete 2	asn,ser,tyr,asn,fatty_acid,mal,inactive	0	0	0	1
Incomplete 3	ser,asn,asn,tyr,asn,fatty_acid,mal	0	2	0	0
Incomplete 4	thr,ser,tyr,asn,fatty_acid,mal	0	1	0	0
Incomplete 5	ser,asn,gln,asn,tyr,asn,fatty_acid,mal,inactive	0	0	1	0
Incomplete 6	ser,asn,pro,gln,asn,tyr,fatty_acid,mal	0	1	0	0
Incomplete 7	thr,ser,glu,pro,asn,tyr,fatty_acid,mal	0	1	0	0
Incomplete 8	ser,glu,pro,asn,tyr,asn,fatty_acid,mal	0	1	0	0
