# Supplementary material for: Phylogenetic Distribution of Secondary Metabolites in the Bacillus subtilis Species Complex
Source: mSystems. 2021 Mar 9;6(2):e00057-21. doi: 10.1128/mSystems.00057-21 (PMC8546965; doi:10.1128/mSystems.00057-21)
Supplement: FIG S5 [file msystems.00057-21-sf005.pdf]

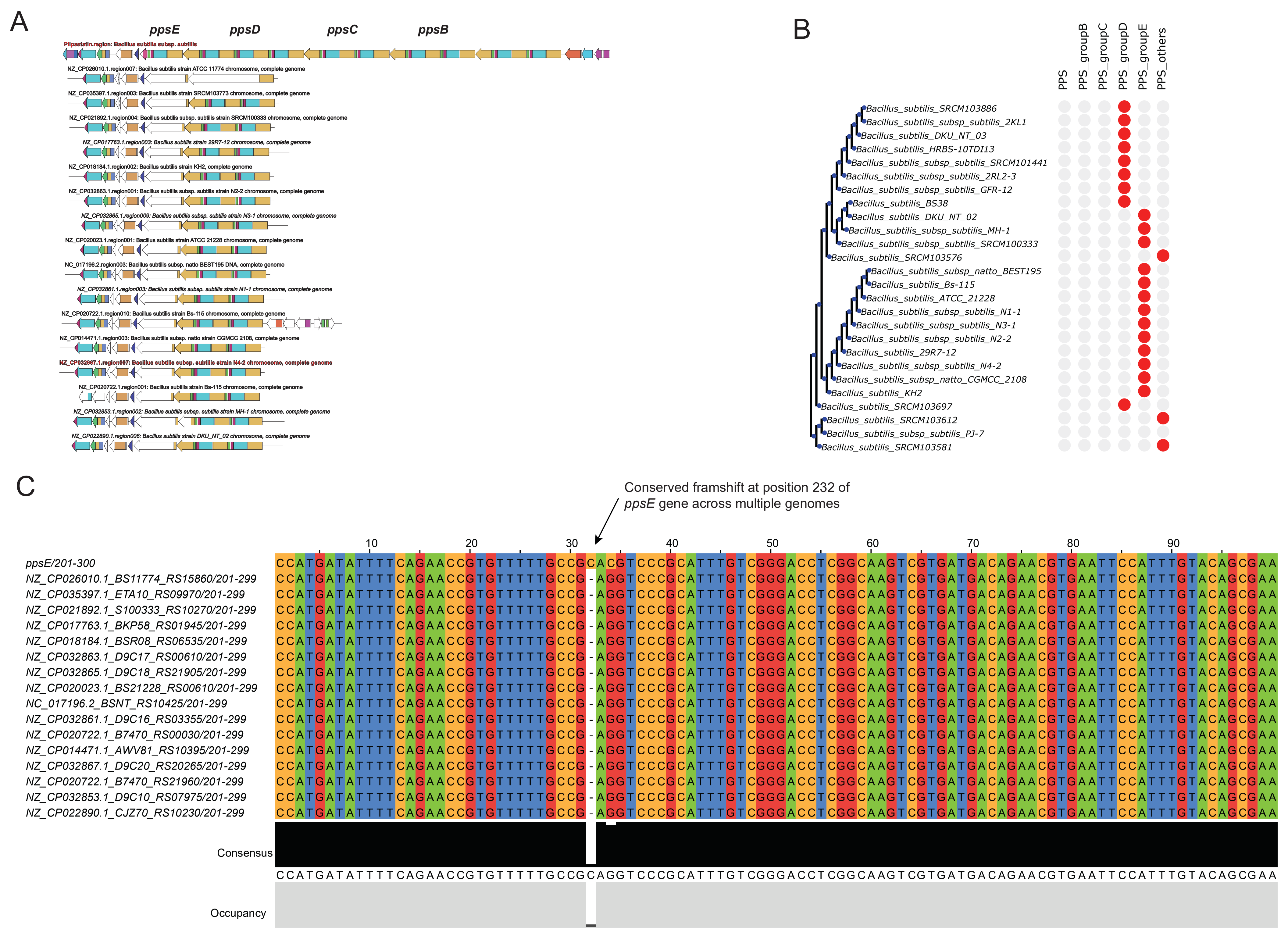
A
ppsE
ppsD
ppsC
ppsB
Plipastatin.region: Bacillus subtilis subsp. subtilis
NZ_CP026010.1.region007: Bacillus subtilis strain ATCC 11774 chromosome, complete genome
NZ_CP035397.1.region003: Bacillus subtilis strain SRCM103773 chromosome, complete genome
NZ_CP021892.1.region004: Bacillus subtilis subsp. subtilis strain SRCM100333 chromosome, complete genome
NZ_CP017763.1.region003: Bacillus subtilis strain 29R7-12 chromosome, complete genome
NZ_CP018184.1.region002: Bacillus subtilis strain KH2, complete genome
NZ_CP032863.1.region001: Bacillus subtilis subsp. subtilis strain N2-2 chromosome, complete genome
NZ_CP032865.1.region009: Bacillus subtilis subsp. subtilis strain N3-1 chromosome, complete genome
NZ_CP020023.1.region001: Bacillus subtilis strain ATCC 21228 chromosome, complete genome
NC_017196.2.region003: Bacillus subtilis subsp. natto BEST195 DNA, complete genome
NZ_CP032861.1.region003: Bacillus subtilis subsp. subtilis strain N1-1 chromosome, complete genome
NZ_CP020722.1.region010: Bacillus subtilis strain Bs-115 chromosome, complete genome
NZ_CP014471.1.region003: Bacillus subtilis subsp. natto strain CGMCC 2108, complete genome
NZ_CP032867.1.region007: Bacillus subtilis subsp. subtilis strain N4-2 chromosome, complete genome
NZ_CP020722.1.region001: Bacillus subtilis strain Bs-115 chromosome, complete genome
NZ_CP032853.1.region002: Bacillus subtilis subsp. subtilis strain MH-1 chromosome, complete genome
NZ_CP022890.1.region006: Bacillus subtilis strain DKU_NT_02 chromosome, complete genome
B
PPS
PPS_groupB
PPS_groupC
PPS_groupD
PPS_groupE
PPS_others
Bacillus_subtilis_SRCM103886
Bacillus_subtilis_subsp_subtilis_2KL1
Bacillus_subtilis_DKU_NT_03
Bacillus_subtilis_HRBS-10TDI13
Bacillus_subtilis_subsp_subtilis_SRCM101441
Bacillus_subtilis_subsp_subtilis_2RL2-3
Bacillus_subtilis_subsp_subtilis_GFR-12
Bacillus_subtilis_BS38
Bacillus_subtilis_DKU_NT_02
Bacillus_subtilis_subsp_subtilis_MH-1
Bacillus_subtilis_subsp_subtilis_SRCM100333
Bacillus_subtilis_SRCM103576
Bacillus_subtilis_subsp_natto_BEST195
Bacillus_subtilis_Bs-115
Bacillus_subtilis_ATCC_21228
Bacillus_subtilis_subsp_subtilis_N1-1
Bacillus_subtilis_subsp_subtilis_N3-1
Bacillus_subtilis_subsp_subtilis_N2-2
Bacillus_subtilis_29R7-12
Bacillus_subtilis_subsp_subtilis_N4-2
Bacillus_subtilis_subsp_natto_CGMCC_2108
Bacillus_subtilis_KH2
Bacillus_subtilis_SRCM103697
Bacillus_subtilis_SRCM103612
Bacillus_subtilis_subsp_subtilis_PJ-7
Bacillus_subtilis_SRCM103581
C
Conserved framshift at position 232 of ppsE gene across multiple genomes
10
20
30
40
50
60
70
80
90
ppsE/201-300 CCATGATATTTTCAGAACCGTGTTTTTGCCGCACGTCCCGCATTTGTCGGGACCTCGGCAAGTCGTGATGACAGAACGTGAATTCCATTTGTACAGCGAA
NZ_CP026010.1_BS11774_RS15860/201-299 CCATGATATTTTCAGAACCGTGTTTTTGCCG-AGGTCCCGCATTTGTCGGGACCTCGGCAAGTCGTGATGACAGAACGTGAATTCCATTTGTACAGCGAA
NZ_CP035397.1_ETA10_RS09970/201-299 CCATGATATTTTCAGAACCGTGTTTTTGCCG-AGGTCCCGCATTTGTCGGGACCTCGGCAAGTCGTGATGACAGAACGTGAATTCCATTTGTACAGCGAA
NZ_CP021892.1_S100333_RS10270/201-299 CCATGATATTTTCAGAACCGTGTTTTTGCCG-AGGTCCCGCATTTGTCGGGACCTCGGCAAGTCGTGATGACAGAACGTGAATTCCATTTGTACAGCGAA
NZ_CP017763.1_BKP58_RS01945/201-299 CCATGATATTTTCAGAACCGTGTTTTTGCCG-AGGTCCCGCATTTGTCGGGACCTCGGCAAGTCGTGATGACAGAACGTGAATTCCATTTGTACAGCGAA
NZ_CP018184.1_BSR08_RS06535/201-299 CCATGATATTTTCAGAACCGTGTTTTTGCCG-AGGTCCCGCATTTGTCGGGACCTCGGCAAGTCGTGATGACAGAACGTGAATTCCATTTGTACAGCGAA
NZ_CP032863.1_D9C17_RS00610/201-299 CCATGATATTTTCAGAACCGTGTTTTTGCCG-AGGTCCCGCATTTGTCGGGACCTCGGCAAGTCGTGATGACAGAACGTGAATTCCATTTGTACAGCGAA
NZ_CP032865.1_D9C18_RS21905/201-299 CCATGATATTTTCAGAACCGTGTTTTTGCCG-AGGTCCCGCATTTGTCGGGACCTCGGCAAGTCGTGATGACAGAACGTGAATTCCATTTGTACAGCGAA
NZ_CP020023.1_BS21228_RS00610/201-299 CCATGATATTTTCAGAACCGTGTTTTTGCCG-AGGTCCCGCATTTGTCGGGACCTCGGCAAGTCGTGATGACAGAACGTGAATTCCATTTGTACAGCGAA
NC_017196.2_BSNT_RS10425/201-299 CCATGATATTTTCAGAACCGTGTTTTTGCCG-AGGTCCCGCATTTGTCGGGACCTCGGCAAGTCGTGATGACAGAACGTGAATTCCATTTGTACAGCGAA
NZ_CP032861.1_D9C16_RS03355/201-299 CCATGATATTTTCAGAACCGTGTTTTTGCCG-AGGTCCCGCATTTGTCGGGACCTCGGCAAGTCGTGATGACAGAACGTGAATTCCATTTGTACAGCGAA
NZ_CP020722.1_B7470_RS00030/201-299 CCATGATATTTTCAGAACCGTGTTTTTGCCG-AGGTCCCGCATTTGTCGGGACCTCGGCAAGTCGTGATGACAGAACGTGAATTCCATTTGTACAGCGAA
NZ_CP014471.1_AWV81_RS10395/201-299 CCATGATATTTTCAGAACCGTGTTTTTGCCG-AGGTCCCGCATTTGTCGGGACCTCGGCAAGTCGTGATGACAGAACGTGAATTCCATTTGTACAGCGAA
NZ_CP032867.1_D9C20_RS20265/201-299 CCATGATATTTTCAGAACCGTGTTTTTGCCG-AGGTCCCGCATTTGTCGGGACCTCGGCAAGTCGTGATGACAGAACGTGAATTCCATTTGTACAGCGAA
NZ_CP020722.1_B7470_RS21960/201-299 CCATGATATTTTCAGAACCGTGTTTTTGCCG-AGGTCCCGCATTTGTCGGGACCTCGGCAAGTCGTGATGACAGAACGTGAATTCCATTTGTACAGCGAA
NZ_CP032853.1_D9C10_RS07975/201-299 CCATGATATTTTCAGAACCGTGTTTTTGCCG-AGGTCCCGCATTTGTCGGGACCTCGGCAAGTCGTGATGACAGAACGTGAATTCCATTTGTACAGCGAA
NZ_CP022890.1_CJZ70_RS10230/201-299 CCATGATATTTTCAGAACCGTGTTTTTGCCG-AGGTCCCGCATTTGTCGGGACCTCGGCAAGTCGTGATGACAGAACGTGAATTCCATTTGTACAGCGAA
Consensus
CCATGATATTTTCAGAACCGTGTTTTTGCCGCAGGTCCCGCATTTGTCGGGACCTCGGCAAGTCGTGATGACAGAACGTGAATTCCATTTGTACAGCGAA
Occupancy
