# Supplementary material for: Phylogenetic Distribution of Secondary Metabolites in the Bacillus subtilis Species Complex
Source: mSystems. 2021 Mar 9;6(2):e00057-21. doi: 10.1128/mSystems.00057-21 (PMC8546965; doi:10.1128/mSystems.00057-21)
Supplement: Reviewer comments [file reviewer-comments.pdf]

AMERICAN SOCIETY FOR MICROBIOLOGY | mSystems®

Dual RNA-seq meta-analysis in *Plasmodium* infection identifies host-parasite interactions

Parnika Mukherjee, Gaetan Burgio, and Emanuel Heitlinger

Corresponding Author(s): Emanuel Heitlinger, Humboldt University, Berlin and Leibniz Institute for Zoo and Wildlife Research, Berlin

Review Timeline:

Submission Date:	February 18, 2021
Accepted:	March 4, 2021

Editor: Sergio Baranzini

Reviewer(s): The reviewers have opted to remain anonymous.

Transaction Report:

(Note: With the exception of the correction of typographical or spelling errors that could be a source of ambiguity, letters and reports are not edited. The original formatting of letters and referee reports may not be reflected in this compilation.)

DOI: https://doi.org/10.1128/mSystems.00182-21

Dear Dr. Sergio Baranzini,

Reviewers' comments received for our previously submitted manuscript "mSystems00845-20 (Publicly available transcriptomes provide the opportunity for dual RNA-Seq meta analysis in *Plasmodium* infection)" and our responses are given in detail here. Where possible, we mention line numbers, figure and table numbers.

Editor's and Reviewer's comments are indented and quotes from the manuscript are in italics.

Editor's summary:

> [...]
>
> While your paper addresses an interesting question, the reviewers stated several concerns about your study and did not recommend publication in mSystems at this moment. In particular, please note the following. While both reviewers see merit in the newly formatted submission as research article, important issues are still raised that will need to be addressed in order for this article to be considered further. For example, the suggestion of reviewer #3 to build a gene regulatory network using additional sources should be considered.

We felt encouraged to resubmit the paper by the reviewers' suggestions and what we perceived as a generally positive evaluation of our study. We addressed all the comments, provide additional analyses (including an analysis inspired by reviewer #3's comment on regulatory networks) and substantially expand our results and conclusions.
A marked-up manuscript produced automatically by a word processor is provided along with these responses to the reviews.

Reviewer comments:

Reviewer #1 (Comments for the Author):

Major Comments:

> -Line 227-228: what do the authors mean with "suitable blood stage runs are identified ..."?

Response: By "suitable blood stage runs", we meant potentially suitable runs/samples from studies that investigated the blood stage of *Plasmodium* life cycle. We now more explicitly note that, in principle, suitable studies provide

“potentially suitable” runs/samples. See Section heading “*Blood and liver samples from different studies and host parasite systems are potentially suitable for dual RNA-Seq analysis*”.

We removed the paragraph that is quoted by the reviewer here starting with "Suitable blood stage runs are identified with..." and provided a more explicit evaluation of different thresholds for the detection of host and parasite transcriptomes (not to be confused with the selection of suitable studies with “potentially suitable” runs). This evaluation is presented in the results paragraph “*Evaluation of thresholds on transcriptome representation improve the analysis of co-regulated gene-expression*“

> -Line 234-244: the authors should be more specific. Are the authors referring to the multi-layer network approach? If yes, the authors should clearly say so. They also should introduce the other approach (that merges all data before computing correlation). It seems that authors are also referring to Fig. 3 in this paragraph? If yes, they should mention it.

Response: This paragraph was referring to the heatmap, wrongly labelled as “Fig 3”. The correct labelling should have been "Fig 2".

In our revision, Fig 2 now shows the heatmap and Fig 3B now shows (as lines 282-283 mention) *"two different but interlinked workflows to reconstruct a consensus network of expression correlation".*

> -Fig. 2: labels in the different panels of the figure are missing. What is panel A and B?

Response: Both previously mislabelled figures now have correct labelling of their panels.

> -Line 250-255: is the order of Fig. 2 and Fig. 3 inverted? This paragraph should be in the previous section where Fig. 2 is mentioned.

Response: Yes, this is correct. The order was inverted, we corrected this.

> -Fig 3:
> • authors focused on the parasite coverage but what about the host coverage? The heatmap should include "Median_host%" information as well.

Response: We previously referred to the percentage of the reads mapping to parasite (mapping to host would be 100 – parasite %). We agree with the reviewer that the proportions of the respective transcriptomes covered are more meaningful metrics (as also used in our thresholds). We changed the figure to represent the median percentage of parasite and host transcriptomes covered (as also used in the thresholds for selecting runs).

> • The authors should try the Jaccard index or a similar metric to compute the

distance (i.e. overlap) between networks instead of raw overlapping. It would be interesting to check if the clustering pattern is the same.

Response: This is a brilliant suggestion. We have added a heatmap with the same datasets as Fig 2C showing the Jaccard index. We now also use the Jaccard index in our standardized selection procedure of the most suitable thresholds for transcriptome representation.

• Authors should explain how the studies included in the heatmap were selected among the 63 potential studies they started with.

Response: We updated our analysis to now provide an unbiased analysis of all potentially suitable runs from all suitable studies. As host-parasite co-expression analysis would need host and parasite detectable transcriptome expression, we analysed studies that have at least 50% (intermediate threshold) of host and parasite transcriptomes detected. For each of these studies, we analysed the study without thresholds ("all"), intermediate ("int") and if available, the stringent ("str") thresholds. We have described this method in details in the methods section "*Selection of runs for analysis*" and Fig 2A provides an overview of the workflow for interpretation of the results on heatmaps in the same figure. Altogether, runs from a total of 20 out of the 36 blood stage studies were analysed. Data from 16 studies was excluded as no thresholds were met by any of the runs.

The results of this selection process are in lines 256-263: "*36 studies investigated the blood stages of Plasmodium. 13 of these studies provided more than 5 runs (our criterion for separate analysis) at all thresholds (schematic in Fig 2A) and were analysed as independent datasets. Similarly, 11 studies could be analysed separately with selection of runs at a "stringent" threshold of 70% transcriptome coverage. To make use of runs not meeting thresholds from studies that couldn't be analysed separately, further, 6 runs from 2 studies were pooled into a combined "humanPvivax" dataset and 10 runs from 5 studies into a combined mouse dataset at intermediate thresholds. The combined mouse dataset comprised 4 runs from 2 P. berghei studies, 5 runs from 2 P. chabaudi studies and 1 run from a P. yoelii study.*"

-Line 262-264: • again, what were the criteria for selecting the "overall" dataset? The authors should explain their rationale. Additionally, the authors should add a supplementary dataset file with the "overall" network (with the 590,000 edges and corresponding correlation scores and p-values) for readers that would like to explore the putative inter-species interactions.

Response: Out of the datasets analysed and represented on the heatmaps (Fig 2), we chose those datasets (thresholds on transcriptome representation) from each study that had the highest sum of Jaccard Indices with all other studies. These chosen datasets (analysed individually for our "multilayer network" approach) comprised the "overall" dataset. A total of 915 runs were included in the "overall" dataset, compared to the 749 runs from the previous version of our MS.

The methods pertaining to this are described in section "*Selection of runs for*

analysis" , lines 149-156: *"To compare sub-datasets with runs selected at different thresholds and including all runs without thresholds, we calculated the Jaccard index for every pairwise combination of these datasets.*

The Jaccard Index is defined as $\frac{|A \cap B|}{|A \cup B|}$ *[21], where A and B are the set of bipartite edges in from two datasets. To include the maximum amount of host and parasite data and to use the best dataset from each study, we calculated the sum of all Jaccard Indices from each dataset. We chose the sub-dataset with the highest Jaccard index for a given study for further analysis and concatenated these representative studies into an "overall" dataset to compute possible conserved interactions from all host-parasite systems.*"

Results pertaining to the selection of runs to make the "overall" datasets are in lines 264-268:

"Based on the sum of Jaccard indices (see Methods) of the datasets, we selected a total of 15 sub-datasets maximizing overlap between individual study networks. We concatenated them to construct the "overall" dataset: 4 studies without thresholds ("all"), 7 at intermediate (50%; "int") and 4 at stringent (70% "str") thresholds (marked with an asterix in Fig 2B,C,D). This means that a total of 915 runs were included in the "overall" dataset."

- A network with 590,000 edges is a big network. Too big in the reviewer's opinion. The authors should include a histogram of the associated correlation values as a supplementary figure.

Response: A histogram is provided in the supplement - Supplementary figure S1 and in Fig 4D (a main figure).

- The authors should apply additional filtering steps (overlapping among different model systems, correlation thresholds, etc) to refine the "overall" network in order to increase the precision of the model. I understand that is how you selected the PLP5-ZDHHC4 interaction (found in five of the six individual networks) described in the manuscript.

Response: Yes, this is a good suggestion that we made sure to follow. To systematically reduce the number of edges in the overall network to a smaller set of relevant edges, we defined core networks as described under section "*A "core" network of evolutionarily conserved interactions*" in Lines 314-326 : "*The "overall" network is a highly connected graph with a total of ~3.64 million edges (Supplementary Table S2). Even though this network provides some resolution relative to the ~56 million edges possible in a network of 13986 host and 4005 parasite genes, the resolution of this network might still be improved. We therefore defined a "core" network: using the "overall" network as a scaffold, we extracted edges that were recovered in at least one human study and at least one model organism. Using this definition, the resultant "core" network has 1876 host genes and 2050 parasite genes connected by 15324 edges (Supplementary Table S3). A list of GO terms enriched or depleted in the genes of the "core" network are*

provided in Supplementary Table S4. As expected, many GO terms were the same as in the "overall" network. Most GO terms in the "overall" network were enriched more strongly because of the higher number of genes in that network. However, many GO terms in the "overall" network were broader than in the “core” network. The recovery of more specific functions in the "core" network indicates higher resolution in this network (Table S4)."

-Line 264: do the authors mean Fig. 4?

Yes, apologies for this. We made sure to refer to all figures correctly in the current update of the manuscript.

-Fig 4:
• Information about the units of the y-axis is missing in panel B

Response: The y-axis in the UpSetR plot shows the log10 transformed number of edges in the intersection of studies. We have added this in the figure: "*(Log10) Number of gene pairs in the intersections*"

• Please explain in the figure legend what different edge colors mean. For example, what does overall+4 mean? I guess that you are referring to edges found in four out of seven networks, including the overall network?

Response: Yes, this is correct. It is now added to the figure legend, "*The number of networks an edge is found in is represented by the edge colour.*"

-Line 267-271: please include the accession IDs of the studies you are referring to (i.e. "dominant studies")

Response: We have added the study IDs now: Line 300 : "*... a mouse study (study ID ERP004598 [91]) ...*" and lines 303 : "*... was a dual RNA-Seq study (on monkey, study ID SRP118827 [27])...* "

-Line 279-306: can the authors generate an additional figure for the interaction between enriched processes from host and parasite? An interaction network at the biological process level could offer key information. That could answer a question like: are the host genes involved in "cell-cell adhesion mediated by cadherin" associated with parasite genes involved in a particular process?

Response: We have now added an interaction network of host and parasite GO terms in Fig 7 (main manuscript figure). This figure shows the GO terms for parasite and host genes that interact creating a network of host and parasite GO terms. The figure itself only shows the network associated with adhesion-related host GO terms. Supplementary tables 6A and B contain the full list of associated GO terms for the core and overall networks, respectively.

-Line 307-309: positive or negative correlation?

Response: After revising our construction of the "overall" dataset by including more studies, as described in our response above, we now have edges that were common to a total of 6 studies plus the overall dataset, instead of only 5, as was in the previous version of our MS. Therefore, the results discussed before with pairs PLP5 - zDHHC4 and UIS21 - zDHHC12 are now not the most striking anymore. They are still found in networks from 5 studies. We don't discuss these now as we want to avoid cherry-picking and focus only on the most striking results (by clear criteria: being shared among the highest number of studies).

We have now added information on correlation for the new resulting gene pairs in the revised MS, line 452-455: "*i) negatively correlated (Pearson's rho = -0.26) Kelch13 in Plasmodium with Laminin subunit beta-2 (LAMB2) in the host and ii) negatively correlated (Pearson's rho = -0.33) parasite 26S protease subunit and host LAMB2, both recovered only in mouse and monkey studies.*"

Minor Comments:

> -Code documentation (GitHub README file) could be improved. Please describe the workflow you developed (including information about the order in which the different scripts were used and their output).

Response: The GitHub README was recently modified as well as the comments for most of the scripts in the repository were improved. We also have versioned this code and uploaded it to Zenodo to obtain a static version with a DOI mentioned on the manuscript (https://doi.org/10.5281/zenodo.4535898), we now reference this as version 1.0 of the analysis.

> -Authors should explain the meaning of the two values shown in Table 1 (e.g. 14/855)

Response: We have now added this to the caption of the table: "*Numbers are arranged as number of studies / number of runs*"

> -Table 2: what do yellow and green boxes indicate?

Response: This was mentioned in the table caption and has been improved to say: "*yellow: intermediate threshold, green: stringent threshold*".

> Reviewer 3: I have read with interest this new version of the previously submitted manuscript. The new format as a research paper rather than a review is a major development that identifies and highlights gene clusters of particular interest to help understanding the malaria disease fight between the parasite and the host response. I have also read in detail the authors' responses and actions, concluding that the new analyses provided and the way of carefully selecting the data sets are convincing and have generated an interesting and weighty paper in the field. Importantly, the metanalysis performed by the authors allow to define and limit future experimental

conditions for other authors that would focus to the most interesting interactions between the host and the parasite that could help identifying co-regulation genes during infection and consequently approaching new therapies in such target genes. I found particularly interesting their approach to select a host-data threshold to reduce the background noise of uninfected cells.

We are grateful that the reviewer liked our methodological approach. Upon this positive comment and requests for clarification from our other reviewer we expanded the presentation of this analysis in sections "*Gene co-expression explains gene essentiality*" and "*Interacting parasite processes and host immune response*".

While I am satisfied with the overall conclusions from the results displaying a huge network form the combined approach as shown in Fig 5 with detailed biological processes and GO terms shared between host and parasite datasets I wonder whether this set of genes can be further decoded (from the expression data) into the blood cell types associated with immunity to malaria in the hosts. Is it possible that the immune cell type would be inferred? For instance by performing further multiomic integration and apply some computational genomics algorithms to analyse, for example, differences in expression of gene regulation elements known belonging to different cell type, or differential activity of transcription factors (TF) in them, that could help to build a gene regulatory network for each immune and non-immune study condition based on TF-regulatory element-target gene interactions.

Response: The reviewer generally suggests to validate/compare our results to external data to show that our networks have biological meaning and are coherent with literature. The reviewer specifically mentions constructing a gene regulatory network between TFs and their respective immune cell genes, the goal being to infer immune cell types.

We believe that a gene regulatory network on the host side is beyond the scope of the present paper - we haven't computed host-host correlations to save computational power. However, we devised two different analysis strategies to address this comment:

Firstly, to provide a validation of our networks with independently measured data, we focused on the intra-parasite expression network. We compared parasite-parasite network properties, such as degree and eigenvector centrality to explain gene essentiality.

This analysis has now been added to the paper in section "*Gene co-expression explains gene essentiality*"

Secondly, To address the biological component of the reviewers request, we used the expression of specific markers for immune cell types in malaria (from 113). We

then checked which of these genes are represented in any of the modules of our host-parasite networks.

This analysis is now described in lines 399-447: "*To test whether our networks (gene clusters) are indicative of correlated gene expression originating from specific immune cells, we used immune cell marker genes established in [113]. These marker genes are specific for 6 types of immune cells in the event of a Plasmodium infection in the blood: ... Overall, these results (Supplementary Table S7) give an indication that certain gene expression clusters in our networks might be associated with specific cells of the innate immune response (neutrophils and monocytes).*" Please see Fig 8B and Supplementary table S7 for the immune cell marker genes found in both the core and overall networks.

While the first analysis (gene essentiality in *Plasmodium*) is rather a validation of our approach and results, this second analysis gives structure to our networks and allows to deduce cell-specific responses potentially interesting for immunologists. We hope that both analysis combined are considered suitable by the reviewer to add the required additional insight in our manuscript.

We also added considerable biological detail and discussion by highlighting some of the most striking patterns, gene-clusters and hos-parasite gene-pairs.

February 22, 2021

Dr. Emanuel Heitlinger
Humboldt University, Berlin and Leibniz Institute for Zoo and Wildlife Research, Berlin

Re: mSystems00182-21 (Dual RNA-seq meta-analysis in *Plasmodium* infection identifies host-parasite interactions)

Dear Dr. Emanuel Heitlinger:

Your manuscript has been accepted, and I am forwarding it to the ASM Journals Department for publication. For your reference, ASM Journals' address is given below. Before it can be scheduled for publication, your manuscript will be checked by the mSystems senior production editor, Ellie Ghatineh, to make sure that all elements meet the technical requirements for publication. She will contact you if anything needs to be revised before copyediting and production can begin. Otherwise, you will be notified when your proofs are ready to be viewed.

As an open-access publication, mSystems receives no financial support from paid subscriptions and depends on authors' prompt payment of publication fees as soon as their articles are accepted. You will be contacted separately about payment when the proofs are issued; please follow the instructions in that e-mail. Arrangements for payment must be made before your article is published. For a complete list of **Publication Fees**, including supplemental material costs, please visit our website.

Corresponding authors may join or renew ASM membership to obtain discounts on publication fees. Need to upgrade your membership level? Please contact Customer Service at Service@asmusa.org.

For mSystems research articles, you are welcome to submit a short author video for your recently accepted paper. Videos are normally 1 minute long and are a great opportunity for junior authors to get greater exposure. Importantly, this video will not hold up the publication of your paper, and you can submit it at any time.

Details of the video are:

· Minimum resolution of 1280 x 720
· .mov or .mp4. video format
· Provide video in the highest quality possible, but do not exceed 1080p
· Provide a still/profile picture that is 640 (w) x 720 (h) max

We recognize that the video files can become quite large, and so to avoid quality loss ASM suggests sending the video file via https://www.wetransfer.com/. When you have a final version of the video and the still ready to share, please send it to Ellie Ghatineh at eghatineh@asmusa.org.

Thank you for submitting your paper to mSystems.

Sincerely,

Sergio Baranzini
Editor, mSystems

Journals Department
American Society for Microbiology
1752 N St., NW
Washington, DC 20036
E-mail: peerreview@asmusa.org
Phone: 1-202-942-9338

Supplemental Table 2: Accept
Supplemental Table 1: Accept
Supplemental Table 3: Accept
Supplemental Table 6: Accept
Supplemental Table 4: Accept
Supplemental Figure 1: Accept
Supplemental Table 5: Accept
Supplemental Table 7: Accept
